# A Novel Orthoplastic Reconstruction of Relapsed Clubfoot With Total Ankle Arthroplasty

**DOI:** 10.7759/cureus.44796

**Published:** 2023-09-06

**Authors:** Arman J Fijany, Sofia E Olsson, Bijan K Givechian, Ilana Zago, Anthony E Bishay, Thomas Troia, Trevor S Page, Alexander Barnett, Michael W Downey, Maxim Pekarev

**Affiliations:** 1 Plastic Surgery, Vanderbilt University Medical Center, Nashville, USA; 2 Plastic Surgery, Anne Burnett Marion School of Medicine, Texas Christian University, Fort Worth, USA; 3 Plastic Surgery, Yale School of Medicine, New Haven, USA; 4 Neurosurgery, Vanderbilt University School of Medicine, Nashville, USA; 5 Plastic Surgery, Rosalind Franklin University of Medicine and Science, North Chicago, USA; 6 Podiatry, John Peter Smith Health Network, Fort Worth, USA; 7 Trauma and Reconstructive Surgery, Precision Orthopedics and Sports Medicine, Fort Worth, USA

**Keywords:** ankle arthritis, reconstructive microsurgery, limb salvage, orthoplastic surgery, congenital clubfoot, total ankle arthroplasty

## Abstract

Congenital clubfoot is addressed in infancy and rarely persists into adulthood. Ankle arthroplasty is an increasingly popular surgical intervention for patients with ankle arthritis since it allows a natural ankle range of motion and completely replaces a degenerative hindfoot. Here, we describe the first successful total ankle arthroplasty (TAA) for a patient with previously treated congenital clubfoot that reverted later in life. To address the patient’s poor soft-tissue integument and reduce the likelihood of post-surgical complications, a perioperative latissimus muscle-free flap was performed. This two-staged, novel orthoplastic intervention addressed our patient’s ankle issues and appears to be a viable option for clubfoot patients.

## Introduction

Congenital talipes equinovarus (CTEV), also known as clubfoot, is a congenital deformity involving an adducted, supinated, varus foot positioning [1]. This deformity may be associated with other congenital abnormalities, such as ventricular septal defect, cleft lip/palate, spina bifida, and hypospadias, but it is often idiopathic [2]. With a prevalence of approximately 1 per 1000 live births, CTEV is a relatively common congenital disorder [3]. The condition is thought to have multifactorial etiology, with genetic and environmental factors, including maternal diabetes and smoking status having a significant association [4]. It is suggested that vascular disruption is a possible mechanism of clubfoot formation, which may partly explain the vascular anomalies observed in this condition. Additionally, a relationship between congenital arterial malformations and clubfoot has been described with cigarette smoke exposure in utero [5-7]. Moreover, genetic factors such as the PITX1 and TBX4 transcriptional pathways, playing critical roles in the development of the lower extremities, have also been implicated [8,9]. Due to its multifactorial etiology, without a single phenotypic driver of deformity, treatment options for CTEV also vary and are patient-specific.

With the ability to assess for CTEV on fetal ultrasound, the patient’s treatment can begin immediately after birth [10]. However, correction during childhood can still necessitate continued treatment throughout adulthood, especially with the persistence of deformities, pain, and arthritis [11]. Conservative treatment may involve a functional rehabilitation approach or the Ponseti method, involving a progressive series of lower-extremity casting [12]. Both methods entail a series of lower-extremity positional corrections over time until the desired positioning is achieved. Surgical intervention may be indicated if conservative treatment is insufficient, though those treated for congenital clubfoot rarely need intervention in adulthood [13]. Of note, adults previously treated for congenital clubfoot commonly have asymptomatic talar hypoplasia and an associated decreased ability to dorsiflex the ankle [10]. Persistent hindfoot symptoms in adults previously treated for congenital clubfoot often occur due to poor surgical technique leading to subsequent, progressively worsening ankle instability. Adults tend to tolerate under-correction better than overcorrection, with the latter being much more challenging to manage surgically [14]. Therefore, variability in surgical quality highlights an area of potential clinical investigation to improve long-term outcomes following surgical corrections of clubfoot.

Poor primary correction of congenital clubfoot can present in adulthood with pain, poor mobility, degenerative ankle changes, flatfoot, severe ligamentous laxity, and an inability to wear most conventional shoes [15,16]. These individuals also have progressively worsening foot function and limited surgical treatment options, often affecting quality of life. Current popular treatments for symptomatic overcorrected and undercorrected CTEV include ankle arthrodesis (AA) and osteotomies [17]. Abnormally lateral placement of the calcaneus concerning the talus, rearfoot valgus, and forefoot abduction are common signs of an overcorrected clubfoot [18,19]. AA results in locking the foot in a dorsiflexed position via fusion of the tibiotalar joint [17]. AA disrupts the natural biomechanics of gait and can eventually lead to ipsilateral arthritis of the hip, knee, and back [20]. Moreover, forefoot adduction and supination are among the most common persistent deformities resulting from an initial undercorrection of clubfoot [18]. Current literature supports the double-column osteotomy, showing significant improvement among patients undergoing correction of residual adduction, cavus, and rotational deformities [21]. For patients with an incongruent ankle joint; however, there is a high risk for osteotomy failure, often necessitating additional surgical intervention [22].

Additionally, soft tissue contractures requiring further soft tissue correction often complicate residual clubfoot deformity. In adults with previously corrected clubfoot, equinus contracture is another common cause of recurrent ankle issues and is usually addressed surgically [23]. Furthermore, an anterior tibialis tendon transfer may also be indicated, as it can improve foot adduction and prevent dynamic supination [24]. Despite these additional measures, outcomes for those with relapsed clubfoot remain poor [25]. Overall, there is a clear need for an innovative procedure to address clubfoot relapse.

While AA and osteotomy procedures are popular interventions for recurrent clubfoot issues in adulthood, total ankle arthroplasty (TAA) has not yet been reported in the literature as a treatment option for these patients [16]. Historically, TAA has been associated with worse outcomes and increased complications compared to AA [26,27]. Importantly, recent generations of TAA prostheses have shown significantly improved results due to improved operative planning, patient-individualized implant design, and auxiliary balancing procedures [26,28-32]. TAA also preserves the natural biomechanics of gait in the limb, which can be more appealing for patients who wish to remain physically active or those with higher functional demands [33]. TAA can even address severe ankle incongruity - a condition for which osteotomy and AA often have poor results [34]. Although these advancements in TAA have improved outcomes in recent years, the soft tissue environment surrounding the ankle remains problematic, serving as a restricting factor for many TAA patients. For patients with a previous history of ankle trauma or surgery, including previous surgical correction of CTEV, the compromised nature of the soft tissue around the ankle is often a contraindication for TAA [33,35].

Despite the mentioned instances of soft tissue compromise, reconstructive microsurgical procedures, such as muscle-free flap transfer for prosthetic joint salvage or soft tissue compromise, have been well-established in the literature to address these issues [31,36]. Both soft tissue discrepancies and irregularities of the bony components within the ankle have been cited as common causes of TAA implant failure [37,38]. In particular, the dorsal component of the ankle - which has a single blood supply from the anterior tibial artery - is often a problem area for soft tissue complications [29]. A muscle-free flap can transfer healthy soft tissue, obliterate dead space, and provide a reliable blood supply to this area of concern, thereby improving joint function, tissue homeostasis, and healing processes [39]. The latissimus dorsi (LD) is considered one of the workhorse flaps for lower extremity reconstruction [40].

Patient selection is critical to a beneficial outcome, and absolute contraindications to TAA exist, such as active infection, diabetic neuropathy, and peripheral vascular disease [41-44]. The most significant independent risk factors for TAA failure include age over 70 years and primary and post-traumatic osteoarthritis [45]. Concerning TAA as a treatment option for clubfoot, preoperative subtalar mobility, abnormal subtalar joint morphology, and ankle deformities may exist in adult clubfoot patients that would present a challenge to successful operation and patient satisfaction [33,44,46]. However, the literature has described TAA as offering improved foot function, increased range of motion, and joint mobility while decreasing pain with good long-term results and a mean success rate of up to 90% [26,45]. TAA can, therefore, significantly increase a patient’s quality of life, especially for those patients who have difficulty with activities of daily living and desire the ability to participate in low-impact exercise with little to no pain.

We hypothesize that TAA with a staged, perioperative muscle-free flap microsurgical procedure can be an aggressive, safe, orthoplastic intervention for patients with ankle arthritis in adulthood due to previously treated clubfoot. To our knowledge, there are no reports of TAA being performed for patients with congenital clubfoot described in the literature. This case report will add to the evidence on operative procedures utilized for adult clubfoot patients and represent a successful TAA for an adult patient who underwent multiple surgeries to correct the clubfoot deformity during childhood.

## Case presentation

A 43-year-old male with a medical history of gout, hypertension, and congenital bilateral clubfoot was seen for debilitating, chronic right ankle pain and arthritis. His history of multiple surgeries to the same area resulted in compromised skin and soft tissues. The patient reported a 12-year history of multiple tenotomies of the extensor digitorum longus and extensor hallicus longus, capsulotomy of the first metatarsal joint, and contracture releases secondary to the clubfoot issues. Despite extensive treatment, he noted that the right foot and ankle are becoming increasingly painful and endorses pain with walking and activities of daily living. He described his pain as achy and burning in quality, preventing him from falling asleep and waking him up from sleep. He saw several providers who discussed fusion versus amputation to optimize his quality of life, but he did not want to proceed with either option. The patient is otherwise healthy and states his primary concern is his right foot. His goal is to be able to walk and do normal activities of daily living.

On physical exam, the patient had significant trophic changes to the anterior region of his right ankle. Additionally, there was visible bowstringing and contracture of the anterior tibialis tendon. Mild exostosis was noticed on the talonavicular and midfoot, but no pain was noted over these areas. Tenderness was noted upon palpation of the sinus tarsi and during movement of the subtalar joint. Also, there was crepitus during the ankle range of motion, with associated pain throughout the effort. The right ankle showed moderate arthroses with joint space narrowing on imaging. Of note, there was also severe hindfoot valgus and mild flattening of the talus, findings associated with previous overcorrection.

**Figure 1:**
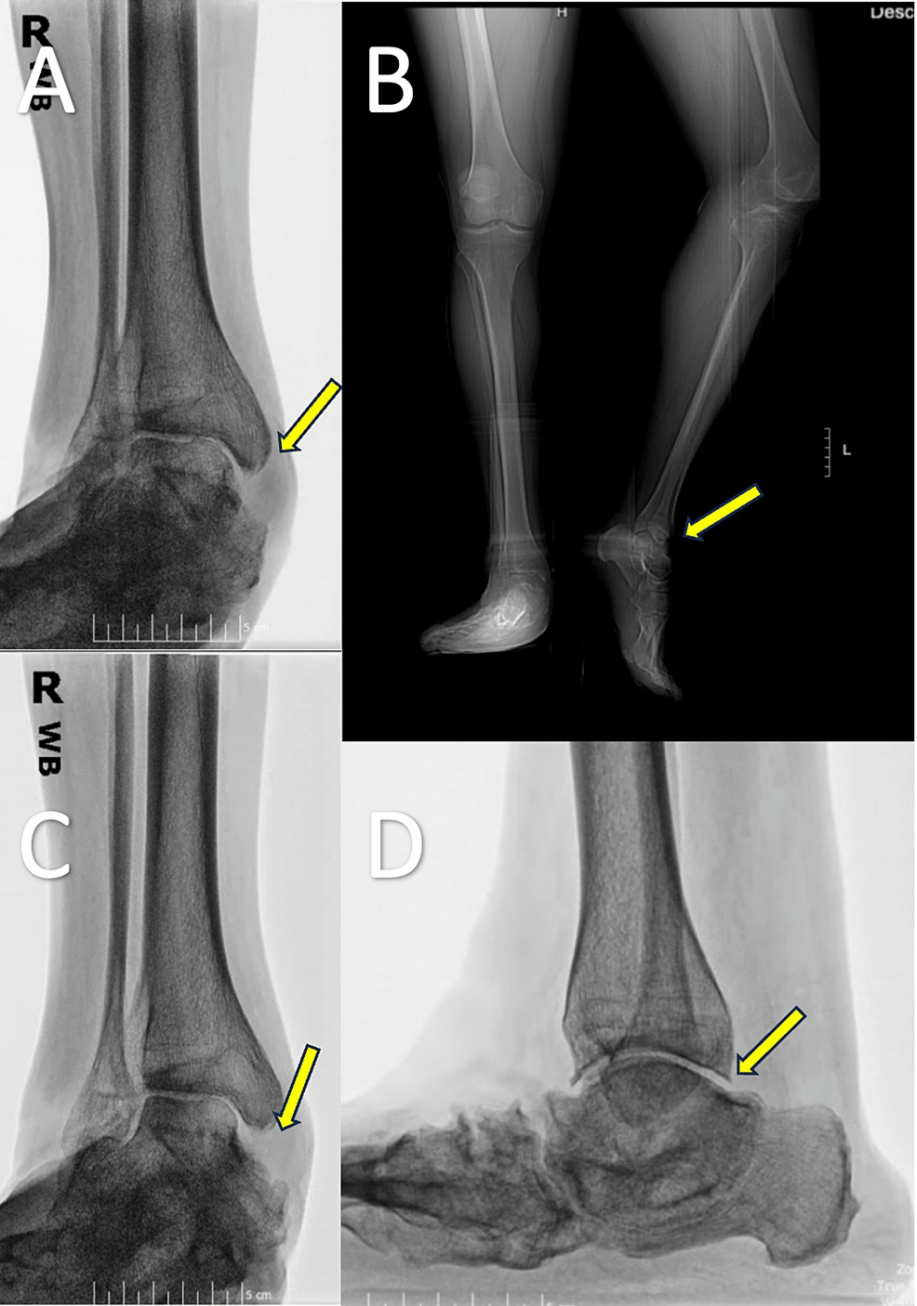
Radiographs of the patient’s left ankle during initial consultation. (A) Anterior-posterior X-ray of the right ankle, showing extensive valgus deformity. (B) Posterior-anterior X-ray of the right ankle, with significant plantar flexion of the ankle. (C) Posterior-anterior X-ray of the right ankle, showing extensive valgus deformity. (D) Mediolateral view X-ray of the right ankle, with significant subtalar joint space narrowing.

The patient was informed that, considering his medical history and deformity, he is a suitable candidate for total ankle replacement. Ideally, he would derive significant benefits from a comprehensive procedure, including calcaneal osteotomy, subtalar joint arthrodesis combined with total ankle replacement, Achilles tendon lengthening, posterior capsulotomy, talonavicular capsulotomy, and, if necessary, tibialis anterior lengthening. Furthermore, due to the poor soft tissue quality in his anterior ankle, it was recommended that he undergo a preventive muscle-free flap protocol. The possibilities of fusion and amputation were discussed with the patient, and he declined both as he would like to maintain his range of motion and avoid amputation at all costs. The patient was informed of risks, benefits, indications, potential complications, and expected rehab course and agreed to proceed with the treatment plan. Radiographs of the patient's right ankle during the initial consultation is shown in Figure 1.

Operative procedure

**Figure 2:**
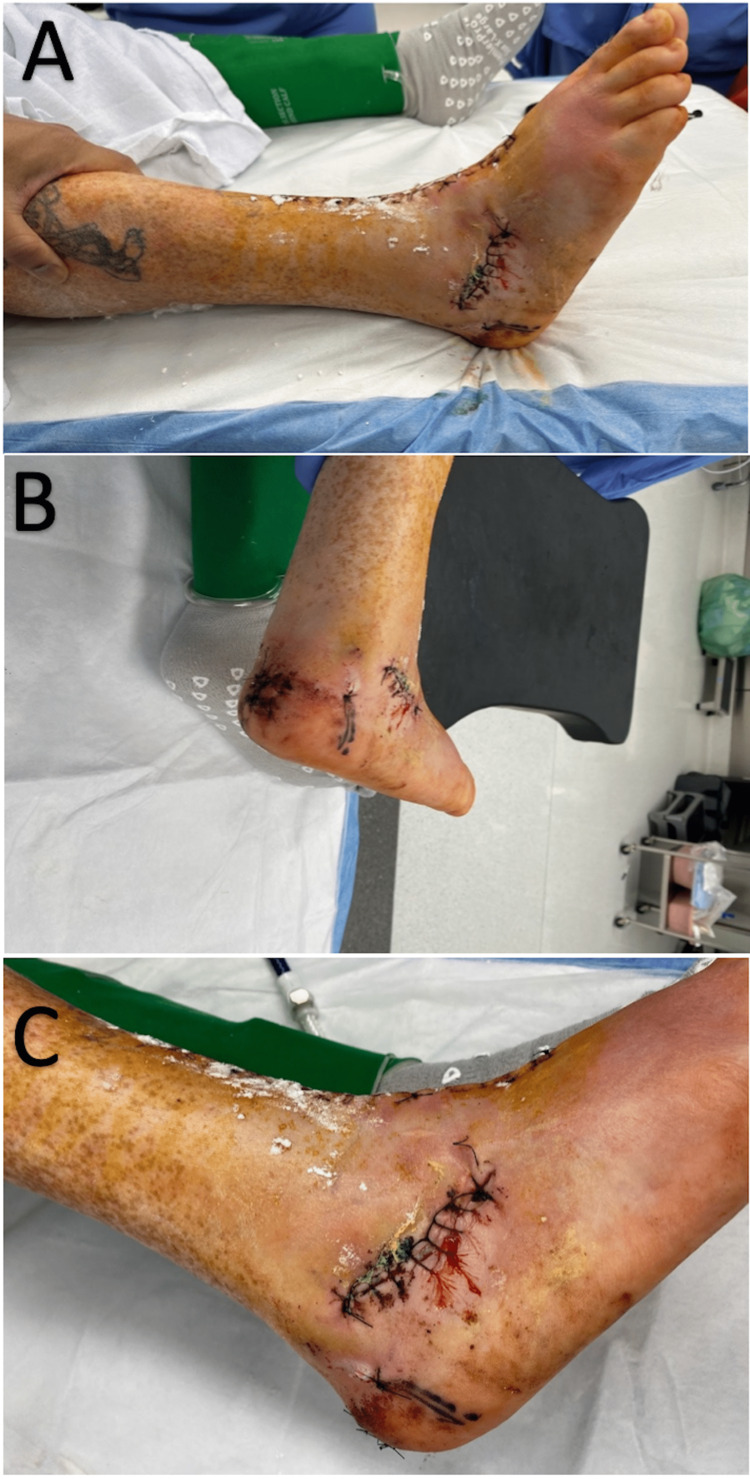
Photographs of the multiple incisions required for TAA. A transverse incision near the medial malleolus was also made but is not shown in the photographs. TAA, total ankle arthroplasty

The patient was brought to the operating room under general anesthesia during the initial procedure. Subtalar joint fusion was then performed with the appropriate joint preparation of the right foot. Afterward, a minimally invasive calcaneal osteotomy was performed and pinned across the subtalar joint. An anterior ankle incision was then made, with careful attention to avoid the anterior tendons and neurovascular bundle. The Prophecy^TM^ INBONE^TM^ total ankle system (Stryker, NJ, USA) was subsequently implanted following the standard technique as per the instructions provided in the surgical guide. After placement of the TAA implant, additional tarsal synovectomy, tibial reinforcement with tibiotalar joint capsulotomy, and talonavicular joint capsulotomy were performed. Radiographs of the patient's right ankle are shown in Figure 2. Of note, the patient had an excellent range of motion of the ankle joint complex, approximately 10° past neutral, so Achilles lengthening was unnecessary.

**Figure 3:**
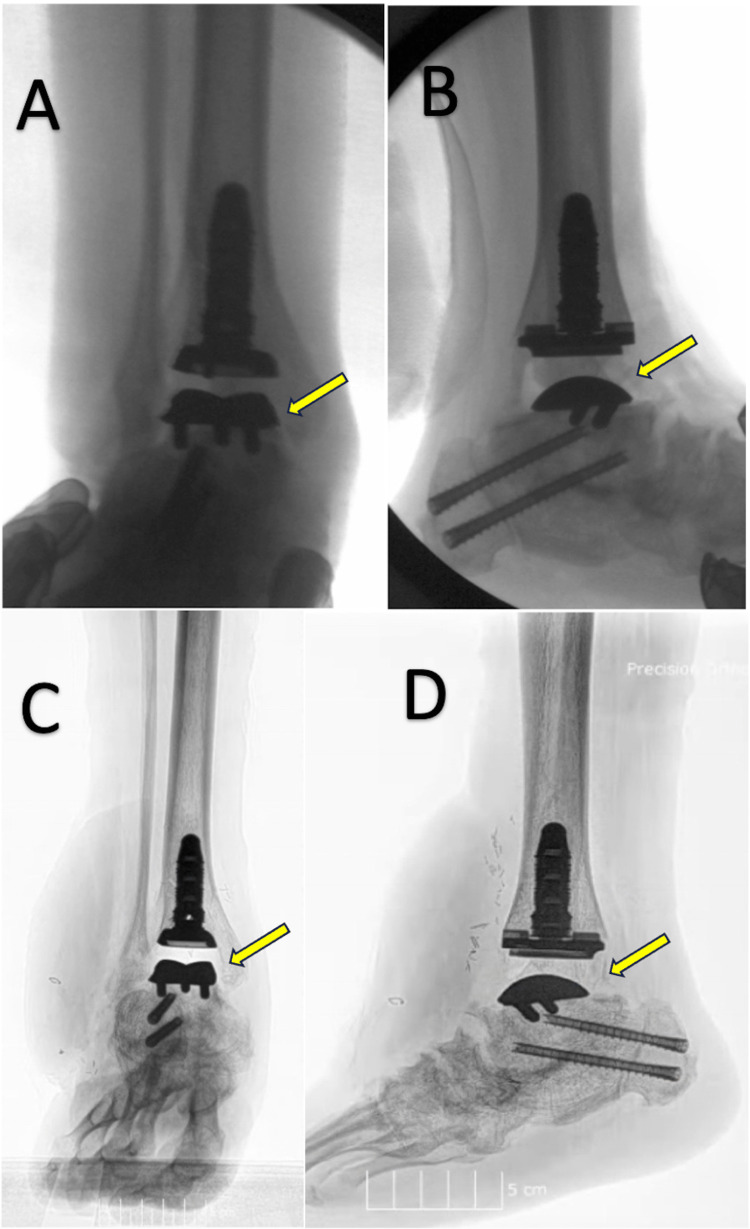
Radiographs of the patient’s right ankle after TAA and free flap microsurgery. (A) Posterior-anterior X-ray view. (B) Mediolateral X-ray view. (C) Anterior-posterior X-ray view. (D) Lateromedial X-ray view. TAA, total ankle arthroplasty

Overall, the patient tolerated the procedure and anesthesia well. He had prompt hyperemic response noted to the digits. The patient was admitted to the hospital overnight for optimization and discharged the following day. The patient was directed to follow a strict non-weight-bearing protocol. Additionally, two pillows were placed underneath the right lower extremity for pain and edema control. Photographs of the patient's right ankle are provided in Figure 3. 

Three days later, the patient returned for the final stage of his orthoplastic intervention. The patient was taken to the operating room for the subsequent microsurgical procedure, and general anesthesia was initiated. Utilizing the prior incision, previous sutures were removed, and a Doppler was used to trace out the anterior tibial vessels. All of the dysvascular tissue was debrided. A plane was then developed between the anterior and extensor hallucis longus to find the anterior tibial vessels using bipolar and sharp dissection. The side branches of the anterior tibial vessels were clipped, and the distal end of the main vessel was ligated to ensure adequate flow. A microscope was brought into the field, and the vessel ends were prepared for microvascular anastomosis by trimming the adventitia. To perform the end-to-side anastomosis, an atraumatic Acland vascular clamp was then placed on the distal end of the main vessel, while the side branch was left open. The anastomosis was completed by suturing the open end of the side branch to the prepared distal end of the main vessel in a precise and secure manner.

Simultaneously, the donor flap was dissected by making a 25 cm incision from the axilla to the posterior iliac crest along the anterior border of the LD muscle. A small skin paddle was included on the mid-axis of the flap to aid in postoperative monitoring. The superficial and deep surfaces of the muscle were dissected using electrocautery to the level of the axilla. The thoracodorsal pedicle was examined together to ensure atraumatic handling. Seven thousand units of heparin were then given at this time. After clamping and ligation proximally, the flap was delivered to the ankle operative site. 

The microscope was then used to trim the adventitia and analogously prepare the vessels to the anterior tibial vessels above. The flap filled nicely, and the distal edges were found to bleed bright red. The site was then dressed with tape. The flap was inserted at the ankle with interrupted 3-0 Monocryl. Doppler was again used to confirm the patency of the vein and artery. 

**Figure 4:**
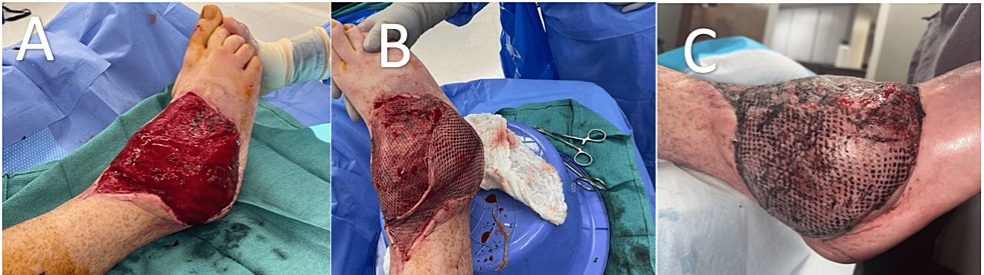
Photographs of the patient’s right ankle after TAA and latissimus free flap transfer. (A) Right ankle after the free flap microsurgery procedure. (B) Right ankle after split-thickness skin graft from the contralateral thigh donor site. (C) Right ankle several weeks after skin graft, showing significant graft take. TAA, total ankle arthroplasty

Three weeks later, a split-thickness skin graft (STSG) procedure was performed, wherein a thin layer of skin, including the epidermis and a portion of the dermis, was carefully harvested from the patient's lateral thigh. The recipient site was prepared by cleaning and debriding nonviable tissue to create an optimal wound bed. The harvested skin graft was meticulously positioned over the wound, ensuring complete coverage without wrinkles or air pockets. The graft was secured in place using sutures, and appropriate dressings were applied to protect the graft and facilitate healing. Post-surgery, the patient was closely monitored to ensure proper graft adherence and wound healing. Representative photographs are presented in Figure 4.

Six weeks post-surgery, the patient reported pain that was 3/10 in intensity, which was not controlled by prescription pain medication. Mild edema was observed, sensation remained intact, and there was slight point tenderness along the muscle flap. Additionally, there were mild restrictions in dorsiflexion, attributed to the bulkiness of the muscle flap. Following this evaluation, the patient started a physical therapy protocol with partial weight-bearing. Six months after surgery, the patient is active, with minimal (ranging from 0/10 to 1/10 in intensity) pain with movement. Ankle range of motion included 15° of dorsiflexion and 35° of plantar flexion. The patient reports that he is *very satisfied* with the procedure and that if he had the choice, he would do the procedure again.

## Discussion

In this patient, we believe that TAA with a staged prophylactic microsurgery was the best option based on the patient’s wishes for a more definitive treatment. While TAA is a relatively more aggressive intervention than other standard treatments for ankle arthritis, such as osteotomy and AA, recent implant design advances have improved TAA outcomes, encouraging its clinical adoption [26,28]. It is important to note that there are still significant risks with such extensive surgery around the ankle, particularly for patients with risk factors such as diabetes, smoking tobacco, hypertension, previous surgeries, and trauma [33,35]. Patient selection is critical - the patient’s desires, risk factors for ankle surgery, and post-surgical compliance must be contemplated methodically before considering this complex, two-staged orthoplastic procedure.

**Figure 5:**
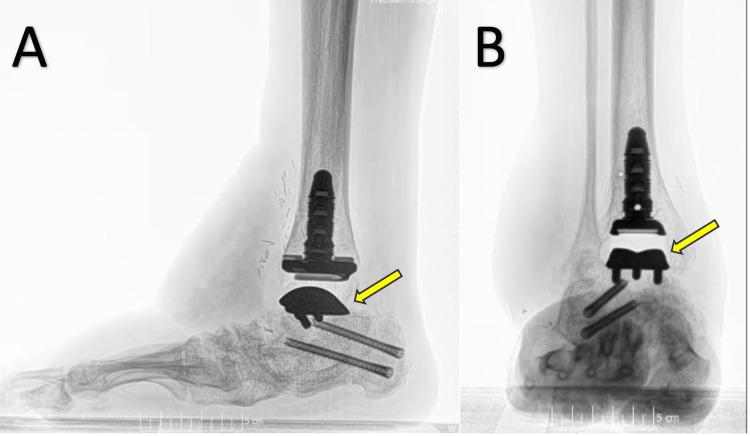
Radiographs of the patient’s right ankle three months postoperatively. (A) Lateral X-ray of the right ankle showing properly placed implants. (B) Anterior-posterior X-ray of the right ankle showing properly placed implants.

**Figure 6:**
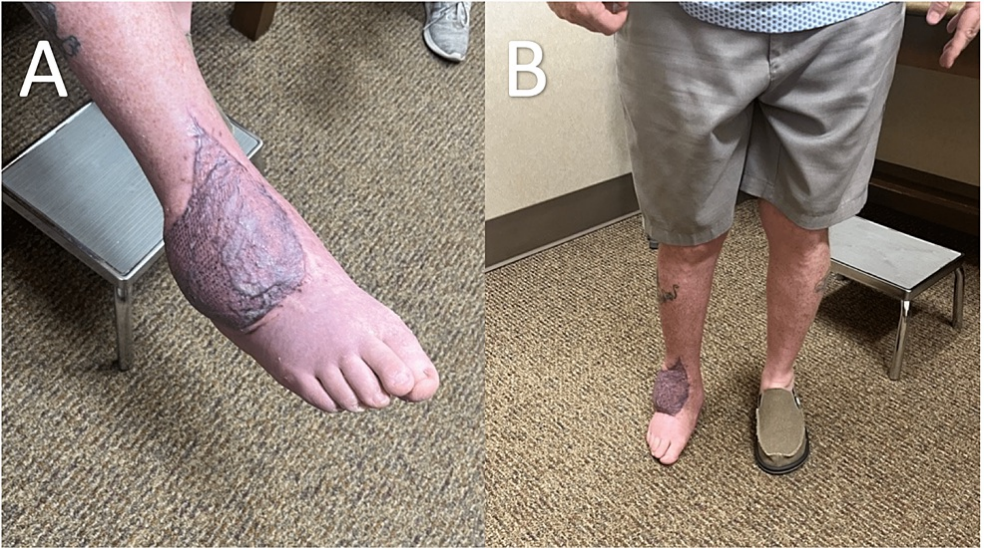
Photographs of the patient’s right ankle three months postoperatively.

Most individuals with congenital clubfoot undergo conservative therapies that are often effective without complications. These patients seldom have a recurrence or persistence of hindfoot dysfunction in adulthood. There are several causes of the persistence of symptoms in adulthood, with overcorrection leading to arguably the most problematic clinical presentation and modest management options. Overcorrection can also present with minimal posterior tibial, flexor digitorum, and flexor hallucis longus muscle activity - requiring extensive physical therapy and surgical intervention to restore normal function [47]. Though we do not know whether our patient’s primary intervention resulted in overcorrection, his substantial valgus heel orientation on physical exam was consistent with previous literature on the adult presentation of overcorrection of clubfoot [18]. While our patient’s presentation was complicated - a right hindfoot with a history of multiple contracture releases, progressive arthritic changes, weak plantar flexion, and substantial scar tissue throughout the ankle from previous surgeries - we observed positive clinical results highlighting the importance of a patient-specific implant design and substantial operative planning. Radiographs and photographs of the patient's right ankle three months postoperatively are shown in Figures 5-6, respectively. 

To our knowledge, no other case reports in the literature overview a TAA for a patient with congenital clubfoot. To this end, some have suggested a hesitancy to undergo TAA in this patient population due to their already diminished preoperative subtalar mobility [16]. Historically, the most common culprit for TAA failure was the prosthesis’ movement or subsidence of the talar component [44]. However, more recent generations of TAA implants have improved the talar component, suggesting increased utility in patients with talar abnormalities [48]. Furthermore, TAA appears to be a superior option in patients with severe ankle dissonance versus AA and osteotomy [49].

It is important to note that a staged microsurgical procedure with a muscle-free flap was essential to address our patient’s compromised soft tissues. A previous history of ankle surgery is often a contraindication to TAA due to an increased risk for postoperative complications; however, we believe a prophylactic, perioperative muscle-free flap transfer can mitigate these risks, as it may help with increasing tissue perfusion for surgical healing and long-term local tissue homeostasis. While this benefit has been shown in high-risk patients undergoing total knee arthroplasty, the literature on a combined orthoplastic intervention for TAA is minimal [50]. Additionally, this procedure was carried out in a low-resource area without access to a large academic center, so a staged procedure was the only available option for the two surgeons and their surgical teams. However, the authors believe that this process could be optimized with a combined procedure in one operating room.

For several reasons, our group selected the LD muscle-free flap versus other potential muscle-free flap options. First, we have successfully used the flap for lower extremity reconstruction and perioperatively to improve soft tissue quality for high-risk TAA patients. Furthermore, the LD muscle offers durability, large size, a slim contour, and minimal donor site morbidity and allows for simultaneous flap and recipient vessel isolation by multiple surgeons [51]. The bulkiness of the LD muscle can be used to address areas of dead space in the anterior ankle. However, it should be noted that the muscle is expected to atrophy over time [52]. Additionally, the size match of the free flap is essential for the patient to wear standard shoes comfortably. While it is true that the LD flap limits the range of motion, our patient was noted to have poor integument anteriorly at the time of consultation and the decision was made to use the bulky LD flap. Although flap options like the radial forearm or anterolateral thigh flap may still be viable choices, it's essential to balance the need for pliability with the need for sufficient coverage and protection.

Amputation is often recommended for patients with end-stage ankle arthritis and compromised soft tissue integument from previous ankle surgeries. This option often relieves pain but does not allow for low-impact physical activity like the two-staged orthoplastic intervention described in the present case study. We believe that a TAA coupled with a perioperative muscle-free flap can be utilized for adults with previously treated congenital clubfoot. This procedure could also be a functional limb salvage option for patients with other vascular or soft tissue compromise sources. A more extensive case series would be required to determine the outcomes of this procedure for patients with different risk profiles.

## Conclusions

For the few patients with previously treated congenital clubfoot who present with ankle dysfunction secondary to overcorrection as a child, surgical options are challenging and not well-established in the literature. Here, we describe a successful joint reconstruction with TAA and concurrent improvement of compromised soft tissues with a perioperative, prophylactic LD muscle-free flap. To our knowledge, there are no other cases of TAA in the literature for a patient with congenital clubfoot.
